# Pre‐saccadic shifts of attention in individuals diagnosed with schizophrenia

**DOI:** 10.1002/brb3.3466

**Published:** 2024-03-07

**Authors:** Matthew Lehet, Martin Rolfs, Jacqueline Bao, Jessica Fattal, Katharine N. Thakkar

**Affiliations:** ^1^ Department of Psychology Michigan State University East Lansing Michigan USA; ^2^ Department of Psychology Humboldt University Berlin Germany; ^3^ Department of Psychology Northwestern University Evanston Illinois USA; ^4^ Psychiatry and Behavioral Medicine Michigan State University College of Human Medicine East Lansing Michigan USA

**Keywords:** corollary discharge, pre‐saccadic attention, psychosis, schizophrenia

## Abstract

**Introduction:**

Pathophysiological theories of schizophrenia (SZ) symptoms posit an abnormality in using predictions to guide behavior. One such prediction is based on imminent movements, via corollary discharge signals (CD) that relay information about planned movement kinematics to sensory brain regions. Empirical evidence suggests a reduced influence of sensorimotor predictions in individuals with SZ within multiple sensory systems, including in the visual system. One function of CD in the visual system is to selectively enhance visual sensitivity at the location of planned eye movements (pre‐saccadic attention), thus enabling a prediction of the to‐be‐foveated stimulus. We expected pre‐saccadic attention shifts to be less pronounced in individuals with SZ than in healthy controls (HC), resulting in unexpected sensory consequences of eye movements, which may relate to symptoms than can be explained in the context of altered allocation of attention.

**Methods:**

We examined this question by testing 30 SZ and 30 HC on a pre‐saccadic attention task. On each trial participants made a saccade to a cued location in an array of four stimuli. A discrimination target that was either congruent or incongruent with the cued location was briefly presented after the cue, during saccade preparation. Pre‐saccadic attention was quantified by comparing accuracy on congruent trials to incongruent trials within the interval preceding the saccade.

**Results:**

Although SZs were less accurate overall, the magnitude of the pre‐saccadic attention effect generally did not differ across groups nor show a convincing relationship with symptom severity. We did, however, observe that SZ had reduced pre‐saccadic attention effects when the discrimination target (probe) was presented at early stages of saccade planning, when pre‐saccadic attention effects first emerged in HC.

**Conclusion:**

These findings suggest generally intact pre‐saccadic shifts of attention in SZ, albeit slightly delayed. Results contribute to our understanding of altered sensory predictions in people with schizophrenia.

## Graphical abstract

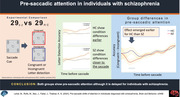

## 1 INTRODUCTION

Mechanistic theories of schizophrenia (SZ) symptoms posit abnormalities in prediction formation and the use of predictions to interpret the causes of input, which ultimately shapes perception and understanding of the world (Fletcher & Frith, 2009; Gray et al., 1991; Hemsley, 1987; Sterzer et al., 2018). An alteration in so‐called predictive processing is argued to underpin the divorce from consensus reality that characterizes the psychotic symptoms of the illness. Predictions are formed on the basis of many different types of information and over many different timescales, from regularities formed over years to sensorimotor predictions about imminent actions on the millisecond scale (Clark, 2013). These sensorimotor predictions are formed on the basis of motor‐related signals that influence sensory processing (corollary discharge signals; CD; Crapse & Sommer, 2008). Predictions based on CD serve several important functions—one of which is to support a subjective sense of agency that occurs when a predicted sensation aligns with actual information from sensory afferents (Haggard, 2017). A disordered sense of agency is thought to underpin many of the symptoms of SZ (Kendler & Mishara, 2019)—particularly the positive symptoms (e.g., hallucinations and delusions)—and alterations in the sensory predictions of ongoing motor programs have been proposed to be one mechanism contributing to these agency disturbances (Feinberg, 1978). Indeed, there is empirical evidence for a reduced influence of sensorimotor predictions in individuals with SZ within multiple sensory systems (Bansal et al., 2018; Pynn & DeSouza, 2013), including in the visual system (Thakkar & Rolfs, 2019; Thakkar et al., 2017).

As the most robust psychophysical and neurophysiological evidence for sensorimotor predictions comes from the literature investigating the influence of saccadic eye movements on visual perception (Pack, 2014; Sommer & Wurtz, 2002, 2008), these systems are well‐suited to serve as a model system to investigate a potential global disruption in sensory predictions during action in individuals with SZ. But altered sensory predictions related to eye movements may also underpin specific symptoms of SZ —particularly those that may be explained in the context of altered allocation of attention, given the tight link between eye movements and visual attention (Bisley & Goldberg, 2003; Moore et al., 2003; Schall, 2004; Sheliga et al., 1995; Shepherd et al., 1986). Now, one function of sensorimotor predictions in the visual system is to proactively shift the focus of attention to a future gaze location (Li et al., 2021; Rolfs & Carrasco, 2012; Rolfs & Schweitzer, 2022; Zhao et al., 2012), thus enabling the prediction of the to‐be‐foveated stimulus (Kroell & Rolfs, 2022). If individuals with SZ have a reduced influence of sensory predictions during action, we would expect their pre‐saccadic attention shifts to be less pronounced. We may speculate that, in this case, sensory consequences of exploratory eye movements would become surprising, capture attention, and therefore be assigned undue significance. Consistent with this notion, clinical descriptions of psychosis have long recognized how—starting in the earliest stages of the illness—aspects of the environment command undue attention and motivational significance. Relatively unimportant objects or events become imbued with meaning and importance (Corlett et al., 2009; Gray, 1998; Kapur, 2003), thereby leading to beliefs or perceptions that are divorced from consensus reality. In this way, altered sensorimotor predictions related to saccades may be linked to aberrant salience assignment and thus psychotic symptoms.

To test this hypothesis, we compared pre‐saccadic attention shifts between individuals diagnosed with SZ and demographically matched healthy controls (HC). In this task, participants were asked to look at a cued location in a stimulus array. A visual probe briefly appeared during saccade preparation, in a location either congruent or incongruent with the location to which they were instructed to look, and participants were asked to make a judgment about that visual probe. Pre‐saccadic attention effects were calculated by comparing probe discrimination accuracy on congruent versus incongruent trials. We hypothesized that pre‐saccadic attention shifts would be less pronounced in individuals diagnosed with SZ and that the magnitude of these pre‐saccadic attention shifts would relate negatively to positive symptom severity. Results may contribute to understanding the broader clinical implications of altered sensory prediction mechanisms associated with eye movements in people with SZ.

## 2 MATERIALS AND METHODS

### 2.1 Participants

Thirty individuals with SZ or schizoaffective disorder and 30 HC were recruited from community advertisements and outpatient mental health facilities. Exclusion criteria are described in Supporting Information Section. Diagnosis was verified using an electronic version of the Structured Clinical Interview for DSM‐5 (Brodey et al., 2016). Two participants (one SZ and one HC) were excluded based on performance (see Supporting Information Section for performance exclusion criteria). Twenty‐seven of the included SZ were using antipsychotic medication, and chlorpromazine equivalent doses (Woods, 2003) were calculated for those participants when possible. See Table 1 for demographic and clinical information for included participants. All participants gave written informed consent approved by the Michigan State University Institutional Review Board and were paid for their participation.

**TABLE 1:** Demographic information.

	HC (*N* = 29)	SZ (*N* = 29)		
	Mean (SD)	Mean (SD)	Statistic	*p*‐Value
Age (years)	38.83 (10.09)	38.38 (11.42)	*t*(56) = .16	.875
Race (white/non‐white)	18/11	19/10	χ^2^ = 0	1
Sex assigned at birth (female/male)	15 F/14 M	10 F/19 M	χ^2^ = 1.12	.289
WTAR	108.48 (8.31)	104.24 (9.45)	*t*(56) = 1.82	.075
CPZ equivalent (mg)		282.78 (275.77)		
SANS total		21.78 (16.94)		
SAPS total		23.78 (22.00)		
Duration of illness (years)		15.63 (10.71)		

Abbreviations: CPZ, chlorpromazine; HC, healthy controls; SANS, Scale for the Assessment of Negative Symptoms total score; SAPS, Scale for the Assessment of Positive Symptoms total score; SZ, individuals with schizophrenia or schizoaffective disorder; WTAR, Wechsler Test for Adult Reading.

### 2.2 Assessments

Clinical symptoms were assessed using the Scale for the Assessment of Positive Symptoms (SAPS) (Andreasen, 1984b) and the Scale for the Assessment of Negative Symptoms (SANS) (Andreasen, 1984a). Estimated premorbid IQ was assessed using the Wechsler Test of Adult Reading (Wechsler, 1999). See Supporting Information Section for details about missing data.

### 2.3 Pre‐saccadic attention task

#### 2.3.1 Apparatus and setup

Participants sat in a dim room with their head stabilized on a chin rest and their eye position recorded with an EyeLink 1000 Plus (SR Research). See Supporting Information Section for additional details.

#### 2.3.2 Design and procedure

**FIGURE 1:**
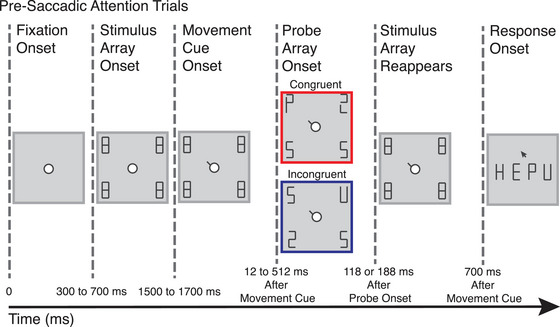
On each trial, participants began by fixating at a central point. Following a variable duration of 300–700 ms, a visual array of four stimuli was presented; 800–1200 ms later, a central movement cue was presented, which instructed the participant to look at the visual stimulus at the cued location. After a short, variable delay, a probe array containing one letter (the probe) was briefly presented (118 or 188 ms) in a location that was either congruent or incongruent with the location indicated by the movement cue. Then, the original stimulus array was presented for a duration that ensured that all trials lasted for 700 ms after movement cue onset. Finally, participants were asked to select the probe stimulus out of four response options using the mouse.

The pre‐saccadic attention task measured the degree to which visual discrimination is predictively enhanced at a future gaze location. The task is described in Figure 1, and additional details are provided in Supporting Information Section. Briefly, on each trial, participants began by fixating at a central point. Following a variable duration of 300–700 ms, a visual array of four stimuli was presented at 5 degrees of visual angle from central fixation; 800–1200 ms later, a central movement cue was presented, which instructed the participant to look at the visual stimulus at the cued location. After a short, variable delay following the presentation of the movement cue, a new visual array (the probe array) briefly replaced the old array. One of the elements of this new visual array was a letter—the probe. The probe could either be at the same location as the instructed saccade location (*congruent*; 25%) or at one of the other three locations (*incongruent*; 25% each) and was presented for either 118 or 188 ms to vary discrimination difficulty. Then, the original stimulus array was presented for a duration that ensured that all trials lasted for 700 ms after movement cue onset. Finally, during the response period, participants were asked to select the probe stimulus out of four response options. The delay between the movement cue onset and the probe onset was dynamically adjusted on the basis of recent saccade latencies to ensure that the probe was presented at roughly equivalent times during the saccade preparation period across participants.

The experiment comprised 288 randomized trials including 256 pre‐saccadic trials and 32 catch trials. Catch trials were included to verify that participants were performing the task as intended. On these catch trials, the probe was presented upon saccade onset and remained on the screen for 188 ms. On congruent catch trials, the probe stimulus was thus present at the location of gaze, enabling an easy discrimination. After finishing the main experiment, participants completed a short posttest that measured peripheral probe detection and the ability to use reliable cues to shift covert spatial attention (see Supporting Information Section).

#### 2.3.3 Data analysis

Analysis of saccade detection, saccade accuracy, and additional details regarding analysis of discrimination accuracy is detailed in Supporting Information Section. Briefly, we used the smoothing method for analysis of response time‐course (SMART) package (van Leeuwen et al., 2019) to examine the time‐course of probe discrimination accuracy as a function of the duration between offset of the probe array and saccade onset—in other words, the time‐course of pre‐saccadic attention shifts. Briefly, the SMART procedure reconstructs a time‐course from data in which each trial contains a single response measure sampled at a certain time (in this case, accuracy at a certain time before saccade onset). First, the data are smoothed to generate individual time‐courses of accuracy, with accuracy values weighted based on the number of data points per participant that contribute at each timepoint. As accuracy scores sometimes approached ceiling levels of performance, we arcsine‐transformed these values (Snedecor, 1956) before submitting them to weighted *t*‐tests at each timepoint to identify accuracy differences between conditions (van Leeuwen et al., 2019) or groups (Shirazi & Huang, 2021). Notably, results were similar when using non‐transformed values (see Supporting Information Section). Finally, a cluster‐based permutation procedure (van Leeuwen et al., 2019) was used to identify timepoints at which accuracy differed significantly between groups or conditions.

We compared groups (SZ vs. HC), conditions (congruent vs. incongruent), and the differences between conditions between groups as a function of time between probe array offset and saccade onset. To assess relationships between clinical symptoms and self‐disturbances across time in SZ, we used a two‐step procedure. First, we conducted a median split on SANS and SAPS scores. For each score, we compared congruent–incongruent accuracy differences between low and high scorers. Within time windows that showed significant differences between high and low scorers, we examined bivariate correlations between symptom scores with individual weighted, congruent–incongruent accuracy differences averaged across that bin. Finally, we compared groups on saccade kinematics (see Supporting Information Section).

## 3 RESULTS

### 3.1 Group and condition effects on accuracy

**FIGURE 2:**
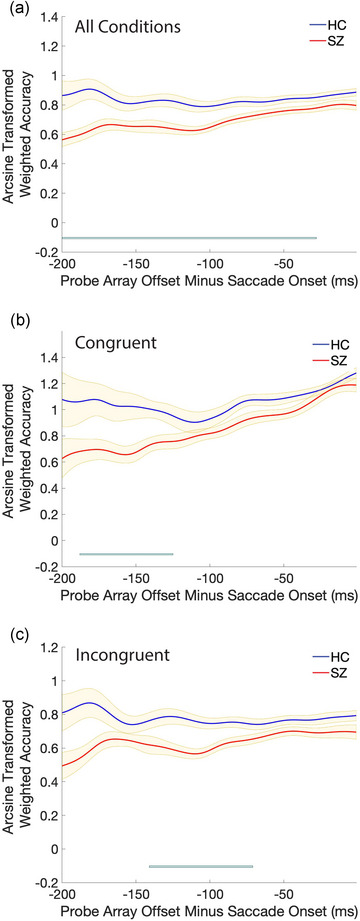
Group differences in arcsine‐transformed discrimination accuracy (A) collapsed across congruency conditions, (B) in only congruent trials, and (C) in incongruent trials. Smoothed weighted averages with the weighted standard error of the mean are plotted for trials where the probe array offset between 200 and 1 ms before the saccade onset. Across all plots, the blue line at the bottom represents clustered significant differences that survived the permutation testing.

**FIGURE 3:**
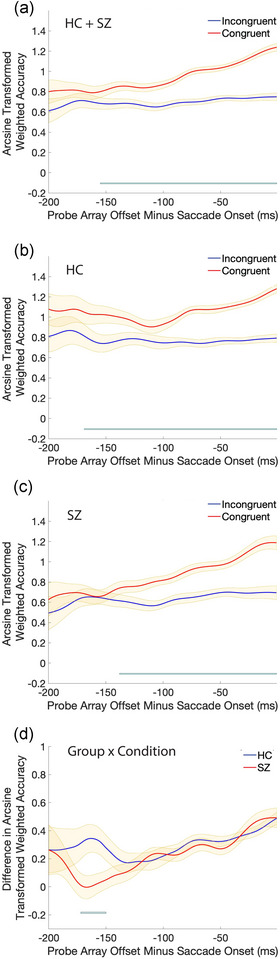
Congruency differences in arcsine transformed discrimination accuracy (A) collapsed across group, (B) in only healthy controls (HC) participants, and (C) in only schizophrenia (SZ) participants. (D) The differences in discrimination accuracy (congruent–incongruent) for the two groups are compared. Smoothed weighted averages with the weighted standard error of the mean are plotted for trials where the probe array offset between 200 and 1 ms before the saccade onset. Across all plots, the blue line at the bottom represents clustered significant differences that survived the permutation testing.

Results are presented in Figures 2 and 3. When accuracy was collapsed across congruency conditions, we observed a main effect of group on accuracy (Figure 2A): HCs were more accurate than SZ except when the probe array offset shortly before saccade onset. On congruent trials (Figure 2B), HC were more accurate than SZ when the probe array offset was between 125 and 188 ms before saccade onset. On incongruent trials (Figure 2C), HCs were more accurate than SZ between 71 and 141 ms before saccade onset. Collapsed across groups, we found a main effect of congruency condition (Figure 3A). While accuracy in the two conditions was initially equivalent, accuracy began to diverge when the probe array offset occurred 155 ms before saccade onset: with shorter intervals between probe array offset and saccade onset, accuracy continuously improved on congruent but not incongruent trials. This robust pre‐saccadic attention effect was observed in both groups, but the congruency effect in HC (Figure 3B) was present 29 ms earlier in the saccade preparation process than in SZ (Figure 3C). This difference was reflected in the analysis comparing congruent–incongruent accuracy differences between the two groups (Figure 3D): SZ showed smaller differences between congruency conditions than HC only when the probe offset was 172–150 ms before saccade onset. This difference between groups in the magnitude of the congruency effect was evident in both probe durations but was statistically significant only in the short probe duration (see Supporting Information Section for a breakdown of results by probe duration).

### 3.2 Relationships between performance and clinical variables

Symptom relationships are described in Supporting Information Section. Briefly, although we observed congruent–incongruent accuracy differences between low and high scorers for SANS and SAPS, results from the median‐split analysis were not confirmed using bivariate correlational analysis. Finally, the magnitude of the congruency effect did not depend on normalized antipsychotic dose.

### 3.3 Posttest analysis

In the posttest (described in Supporting Information Section), we examined how cue congruency and cue reliability affected accuracy across groups when no saccade was made. We found that congruent cues facilitated discrimination in the unreliable cue condition equivalently across groups. Cue reliability did not affect discrimination accuracy.

### 3.4 Saccade kinematics

SZ had slower latencies, smaller amplitudes, increased landing site error, and increased endpoint scatter (Table S1).

## 4 DISCUSSION

In this study, we examined the degree to which programing a saccade leads to a predictive shift in covert attention to the saccade target—pre‐saccadic attention—in SZ and HC. To test these pre‐saccadic attention shifts, we presented a visual discrimination target at one of four locations on the screen, to one of which participants were instructed to direct gaze. Given that the saccade target was not predictive of the probe location, the degree to which discrimination accuracy was enhanced at the saccade target versus elsewhere served as the measure of pre‐saccadic attention. Because theories suggest altered use of predictions to guide behavior in SZ, generally (e.g., Sterzer et al., 2018), and evidence suggests a failure to appropriately predict the sensory consequences of imminent actions specifically (Bansal et al., 2018; Pynn & DeSouza, 2013; Thakkar & Rolfs, 2019), we hypothesized that planning a saccade to a target would not predictively enhance visual processing at that location in SZ to the same extent as HC (i.e., reduced pre‐saccadic attention). Furthermore, we hypothesized that attenuated predictive shifts in attention may lead to visual input that was surprising, thereby being assigned undue significance; in this way, we predicted that reduced pre‐saccadic attention would be related to severity of positive symptoms—symptoms that may have some basis in aberrant salience assignment. These hypotheses were largely unsupported by our data. We observed robust pre‐saccadic attention effects in both groups, such that there was an accuracy advantage when the discrimination target (probe) was presented at the saccade target, which increased as the discrimination target was presented increasingly close to saccade onset. Although SZs were less accurate overall, the magnitude of the pre‐saccadic attention effect (i.e., difference in accuracy between congruent and incongruent trials) generally did not differ across groups nor show a convincing relationship with symptom severity. We did, however, observe that SZ had reduced pre‐saccadic attention effects when the discrimination target was presented at early stages of saccade planning, when pre‐saccadic attention effects first emerged in HC. These findings suggest generally intact pre‐saccadic shifts of attention in SZ, albeit possibly slightly delayed. In the following section, we interpret these results within the literature describing the properties and mechanisms of pre‐saccadic attention shifts and highlight limitations and implications of this work.

Pre‐saccadic attention has been extensively characterized behaviorally; it enhances discrimination, acuity, perception of higher spatial frequencies, and contrast sensitivity at the location of a planned saccade (Hanning et al., 2019; Kroell & Rolfs, 2021; Kwak et al., 2023; Li et al., 2016, 2019; Montagnini & Castet, 2007). Although visual processing is enhanced at the location of the saccade target, visual information is suppressed elsewhere (Buonocore et al., 2017; Khan et al., 2015; Ohl et al., 2017; Shurygina et al., 2021). Along with spatial coupling of oculomotor processes and attentional orienting, there is also temporal coupling: Pre‐saccadic attention increases during the saccade preparation period, peaking in the 75 ms prior to saccade onset (Deubel, 2008; Li et al., 2021; Rolfs & Carrasco, 2012). The spatial and temporal pattern of pre‐saccadic attention effects we found in the current study largely conforms to what has been observed in these prior studies. The exception here is that we did not observe evidence that attention to incongruent locations is suppressed, particularly when the incongruent location is in the same hemifield as—and in close temporal proximity to—the saccade target (Buonocore et al., 2017; Khan et al., 2015; Ohl et al., 2017; Ouerfelli‐Ethier et al., 2023). This process is thought to occur due to lateral inhibition within priority maps, inhibition between hemispheres, and inhibition within the saccade execution system (Ouerfelli‐Ethier et al., 2023). Failure to observe such suppression at locations that are incongruent with the saccade target may be related to the timing of our experiment or the spatial parameters of our experimental stimuli; for instance, the magnitudes of pre‐saccadic attention effects are affected by the spatial frequency of the probe stimulus (Kroell & Rolfs, 2021; Li et al., 2016, 2019).

Pre‐saccadic attention is thought to be enacted via feedback signals between specific neural populations in frontal eye fields (FEF), superior colliculus (SC), and intraparietal sulcus—areas that encode visuospatial priority maps that integrate visual salience and behavioral relevance (reviewed in Bisley & Mirpour, 2019; Cavanagh et al., 2010; Hunt et al., 2019; Jerde et al., 2012; Li et al., 2021; Thompson & Bichot, 2005) and provide attention pointers to relevant retinotopic locations (Cavanagh et al., 2010). CD signals from the SC are relayed to FEF by way of the medial dorsal thalamus, providing information about impending saccades that allows the visual maps in FEF to predictively update ahead of a saccade (Sommer & Wurtz, 2002, 2006, 2008), facilitating visual stability and continuity across eye movements (Rao et al., 2016; Rolfs & Szinte, 2016; Thakkar et al., 2017; Zirnsak & Moore, 2014). The information in these priority maps is relayed to early visual cortex (Thompson & Bichot, 2005), modulating responsiveness of visual neurons preceding a saccade (Mazer & Gallant, 2003; Moore & Armstrong, 2003; Moore et al., 1998; Steinmetz & Moore, 2014). This cortical connectivity transferring information from oculomotor (as in FEF) to visual retinotopic maps (as in V4) constitutes a form of CD that drives pre‐saccadic attention. The relative roles of CD signals sent via cortical‐to‐cortical connections versus subcortical signals relayed to cortex via the thalamus may help explain the current results.

One explanation for the similar performance between groups is that pre‐saccadic attention does not rely on CD. SZs show reduced access to motor plans given reduced integrity of thalamocortical connections that convey CD information from SC to FEF (Yao et al., 2019). This path is a well characterized source of feedback about motor commands and constitutes a transmission pathway for CD signals to the oculomotor planning network (Crapse & Sommer, 2008; Pack, 2014). However, other pathways that update activity in retinotopic maps such as connections between FEF and V4 (Armstrong et al., 2006; Gregoriou et al., 2009; Steinmetz & Moore, 2014) may provide early visual information about the locations of saccade goals identified in the FEF before movement kinematics or efference copies of motor commands are available. To the extent that these connections between cortical regions are equivalent in SZ and HC they could have led to comparable pre‐saccadic attention in both groups.

However, there is a great deal of prior evidence supporting reduced coupling between oculomotor processes and perceptual decisions in individuals with SZ (Bansal et al., 2018; Bansal et al., 2018; Rosler et al., 2015; Thakkar & Rolfs, 2019; Thakkar et al., 2017; Yao et al., 2019). A potential explanation for discrepant results across studies centers on the specific stage of eye movement programing engaged by different tasks. Mounting evidence suggests that pre‐saccadic attention is spatially coupled with the saccade goal, rather than the actual saccade landing position (Deubel & Schneider, 1996; Ditterich et al., 2000; Hanning et al., 2019; Li et al., 2021; Van der Stigchel & De Vries, 2015; Wollenberg et al., 2018, 2020). In contrast, previous studies have shown a reduced influence of saccade programing on perceptual judgments in people with SZ when those judgments rely on an accurate prediction of the precise saccade kinematics—that is, predictions that are dependent on the motor program, rather than the goal. Thus, one interpretation of this study within the broader literature is that individuals with SZ can appropriately form and use predictions related to a motor goal but not the motor plan. To the extent that there are CD signals accompanying a hierarchy of motor‐related signals (from goal selection to execution; Crapse & Sommer, 2008; Subramanian et al., 2019), this broader pattern of results may suggest intact transmission or use of CD signals associated with higher level planning (e.g., goal selection), but not CD signals that are more proximal to movement execution (e.g., efference copies of motor commands).

Now, we did find a small window of time, early in saccade programing, in which individuals with SZ had a reduced pre‐saccadic attention effect compared to controls. This may reflect a slower initiation of pre‐saccadic attention mechanisms among SZ resulting from impaired predictive mechanisms. The temporally selective deficits we see may be explained by research showing that saccade execution facilitates the transfer of visual information at an action‐relevant target from iconic sensory memory into a more robust working memory store (Heuer et al., 2020; Ohl & Rolfs, 2017, 2018). This movement‐related facilitation would be expected to affect performance on the dual task protocol we used here to assess pre‐saccadic attention as participants respond after the saccade. If SZs have weaker or less stable iconic sensory representations after a longer delay between probe offset and saccade onset than HC, there may be less visual information available to be transferred into working memory. A large literature identifying the negative impact of backwards masking on visual detection of targets among SZ supports the idea that early sensory representations are less stable among SZ. The onset of a masker after a target has a larger detrimental impact on early visual representations for SZ than for HC (Green et al., 2011; McClure, 2001). In our paradigm, the stimulus array appears immediately after the probe and could interrupt or integrate with the visual representation of the probe to a greater extent among SZ, leading to weakened sensory representations in the visual system—especially after a longer delay—that are too degraded to transmit to working memory during the saccade.

There are several limitations to these results. First, groups differed in some saccade accuracy metrics, consistent with prior studies (Obyedkov et al., 2019) but see Gooding and Basso (2008), Hutton et al. (1998), Karoumi et al. (1998), and Straube et al. (1999). We identified latency, amplitude, landing site error, and endpoint scatter differences between groups. Although it is possible that differences in kinematics bear on the pre‐saccadic attention findings, we do not think this is likely. Pre‐saccadic attention appears to be linked to an intended location rather than the saccade endpoint (Deubel & Schneider, 1996; Ditterich et al., 2000; Hanning et al., 2019; Li et al., 2021; Van der Stigchel & De Vries, 2015; Wollenberg et al., 2018, 2020). In other words, the redirected attention preceding a saccade is directed toward the saccade goal rather than toward the saccade landing site, reducing the importance of saccade kinematics for pre‐saccadic attention‐based facilitation. Should increased endpoint scatter reflect, in part, a less accurate representation of the saccade goal, then we would expect to see broadly reduced pre‐saccadic attention effects in SZ, which we do not.

Additionally, due to the dynamic timing of probe onset, there are a limited number of trials where the probe offset in the time range where we found meaningful group differences including the differences between congruency conditions (see Figure S6). This means that relatively fewer trials inform the time period of the analysis where we see group differences. In addition, we cannot rule out confounding effects of medication; however, the magnitude of pre‐saccadic attention did not depend on normalized antipsychotic medication dose. Finally, the current sample comprised a stable group of outpatients; altered pre‐saccadic attention may be more apparent during periods of acute psychosis.

## 5 CONCLUSION

To conclude, this work constitutes the first investigation of pre‐saccadic attention in SZ, to our knowledge. Importantly, we developed and tested a rigorous psychophysical dual task protocol that is feasible in clinical population and yields robust pre‐saccade attention effects. Although these predictive shifts in attention are generally intact in people with SZ, we found that they may be slightly delayed relative to controls. These results contribute to our understanding of the nature and specificity of altered sensory predictions in people with SZ.

## AUTHOR CONTRIBUTIONS

**Matthew Lehet**: Writing—original draft; visualization; writing—review and editing; supervision; data curation; formal analysis; methodology; investigation; software; validation. **Martin Rolfs**: Conceptualization; writing—review and editing; methodology; funding acquisition; software. **Jacqueline Bao**: Data curation; investigation; project administration. **Jessica Fattal**: Data curation; investigation; project administration; writing—review and editing. **Katharine N. Thakkar**: Conceptualization; formal analysis; visualization; writing—original draft; methodology; investigation; supervision; project administration; writing—review and editing; funding acquisition; resources.

## CONFLICT OF INTEREST STATEMENT

None of the authors have conflicts of interest.

### PEER REVIEW

The peer review history for this article is available at https://publons.com/publon/10.1002/brb3.3466.

## Supporting information

Supporting Information

Supporting Information

Supporting Information

Supporting Information

Supporting Information

Supporting Information

Supporting Information

Supporting Information

Supporting Information

## Data Availability

The data that support the findings of this study are available from the corresponding author upon reasonable request.
