# Supplementary material for: Pre‐saccadic shifts of attention in individuals diagnosed with schizophrenia
Source: Brain Behav. 2024 Mar 7;14(3):e3466. doi: 10.1002/brb3.3466 (PMC10918725; doi:10.1002/brb3.3466)
Supplement: Supplementary file 1 — Supporting Information [file BRB3-14-e3466-s001.docx]

**Supplemental Methods**

**Exclusion criteria**

Exclusion criteria for both groups included substance use disorder (according to the DSM-5 criteria) within the previous six months, history of neurological disorders, history of head injury with loss of consciousness longer than one hour, and vision that was not normal or corrected-to-normal. HC were excluded if they had a personal history of DSM-5 Axis-I disorders or family history of schizophrenia spectrum disorders or bipolar disorder. Participants were further excluded based on performance, as described below.

**Missing assessment data**

Two SZ did not have SAPS or SANS data collected within six months. Four SZ could not have CPZ calculated due to taking medication with no published CPZ equivalents.

**Design and procedure**

*Apparatus and setup*

Participants were seated in a dimly lit room with their head stabilized on a chin rest 59 cm away from a computer screen. The experiment was presented on a CRT monitor (resolution: 1280 x 960, size 388 mm x 291 mm, vertical refresh rate 85 Hz). During the task, eye position and pupil size of one eye was recorded using an EyeLink 1000 Plus (SR Research, Mississauga, ON, Canada) at a sampling rate set of 1000 hz. Calibration and validation were performed before the practice and after each break in the task. The experiment was written in MATLAB (The MathWorks, Natick, MA) using the Psychophysics (Brainard, 1997) and EyeLink (Cornelissen et al., 2002) toolboxes.

*Procedure overview*

Participants practiced the pre-saccadic attention task. After reaching a threshold level of performance on practice trials, participants completed the pre-saccadic experiment. Following this main experiment, participants completed a short post-test, intended to measure the ability to detect stimuli in the periphery and to use reliable cues to direct covert spatial attention.

*Pre-saccadic attention task*

The experiment included 256 pre-saccadic attention trials (4 probe locations x 4 probe stimuli x 4 cued locations x 2 probe onset durations x 2 probe durations). Each trial began with a fixation check to ensure participants were fixating centrally. During this check, participants fixated a red circle (.15 degrees visual angle radius). Gaze was required to stay within a circle of 2.5 degrees visual angle radius around the center of the circle. Once fixation was confirmed, the center of the fixation turned white. After a variable delay, a four-element visual array consisting of digital number eights (0.52 degrees visual angle wide by 1.05 degrees visual angle high) was presented at four corners of a square around the annulus with their center at 5 degrees of visual angle from central fixation. After a variable delay, a movement cue (a line .12 degrees visual angle in length) was drawn from the fixation point directing the participant to look at one of the four possible cued locations. The onset of the movement cue occurred between 1500 to 1700 ms (sampled from a uniform distribution) after the fixation turned white. The visual array of eights appeared between 1000 to 1200 ms (sampled from a uniform distribution) before the movement cue. After a dynamically adjusted delay following the movement cue (calculated using a procedure that is described later), an array containing the probe replaced the eights. This array contained one digital letter (the probe: either E, U, P, or H) and three digital numbers (either 2 or 5). All elements of the array containing the probe were made up of five line segments and were the same size and displayed in the same locations as the elements in the initial array of number eights. The probe array was presented for either a long (188 ms) or short (118 ms) duration.

Probe array timing was designed to ensure variability in the duration between probe offset and saccade onset and to maximize the number of trials in which the onset of the probe occurred <100 ms before saccade onset (where pre-saccadic attention effects are largest). Since the latency of saccade onset was expected to vary across participants and across trials, the duration between movement cue onset and probe array onset was calculated on a trial-by-trial basis to optimize the onset of the probe array relative to saccade onset. On each trial, delay between movement cue onset and probe array onset was determined based on mean saccade latency and the inter-quartile interval of the distribution of saccade latencies for the most recent 40 saccades. To ensure variability in probe offset relative to movement cue onset, the probe array was presented at one of two equiprobable delays following the movement cue: a short delay or a long delay. These two possible delays (long and short) were dynamically updated across trials. The long delay between probe onset and saccade offset was calculated as the mean saccade latency across the most recent 40 saccades minus one quarter of the inter-quartile range of the saccade latency distribution, minus the probe array duration (118 ms or 188 ms) minus a 71 ms constant. This constant was subtracted to maximize the number of trials in which the onset of the probe occurred <100 ms before saccade onset. The short delay was calculated in two steps. First, 2.5 times the inter-quartile range of the saccade latency distribution the probe array duration, and the 71 ms constant were subtracted from the mean saccade latency. Using this calculated value as one endpoint and the long probe onset delay as a second endpoint, two values were linearly interpolated between these endpoints. The longer of the two linearly interpolated values was used as the short onset delay. This linear interpolation approach was the result of a piloting process aimed at optimizing probe onset timing. Short delays that would cause less than 1 frame delay (11.8 ms) were set to be one frame. Long delays that would cause the probe array to offset after the maximum allowed saccade latency (700 ms) were set to 441 ms for the long duration probe array or 511 ms for the short duration probe array (maximum allowed saccade latency minus probe array duration minus the 71 ms constant). This procedure ensured that on a majority of trials the probe array would offset between 0-100 ms before saccade onset (as shown in **Figure S6**).

In addition to the pre-saccadic attention trials, 32 catch trials were included to verify that participants were performing the task as intended. For catch trials, onset of the probe array was triggered by saccade onset, which was defined as the time at which gaze moved 2.5 degrees from the center of the fixation point. The probe array remained on the screen for 188 ms. On eight of the 32 catch trials the probe location was congruent with the cued saccade direction. Accuracy on congruent catch trials (which are typically an easy discrimination), was used to exclude participants on the basis of performance.

After the practice (procedure detailed below) and after completing each 20% of the experiment, participants took self-timed breaks. They were updated on their progress every 5% of the trials completed. Any trials on which fixation was broken before the movement cue or where a saccade was not made were redone at the end of the experiment.

*Practice design and procedure*

The practice procedure comprised four blocks, designed to get progressively harder and more similar to the real version of the task. The first practice block (10 trials) required participants to make eye movements in the direction indicated by the movement cue. The second practice block (10 trials that included at least 3 congruent trials) is a slowed down version of the full task. Here the initial array (digital number eights) was presented between 300-1000ms after successful fixation, for a duration of 1800-2000ms. Then the movement cue was presented. After a delay of either 300 ms, 400 ms, or 600 ms, the probe array appeared and remained on the screen for 400 ms. Participants were permitted 1500ms to make a saccade. The third practice (14 trials that included at least 3 congruent trials) was a slightly faster version of the task and closer to the real task timings. Here the initial array (digital number eights) was presented between 300-800ms after successful fixation, for a duration of 1800-2000ms. Then the movement cue was presented. After a delay of either 150 ms, 200 ms, or 300 ms, the probe array appeared and remained on the screen for 180 ms. Participants were permitted 700 ms to make a saccade. The final practice (16 trials that included at least 3 congruent trials) had a similar pace to the real experiment. Here the initial array (digital number eights) was presented between 300-500ms after successful fixation, for a duration of 1500-1700ms. Then the movement cue was presented. Like in the real experiment, probe array onset was determined using the distribution of saccade latencies in either the most recent 40 saccades or all available saccades from the first three practice blocks. The probe array was presented for 118 ms and participants had 600 ms to make a saccade.

*Post-test design and procedure*

The post-test consisted of two 16-trial blocks. The spatial layout and temporal dynamics of the task were consistent with the main experiment; however, participants were instructed to fixate centrally throughout each trial for each block. In one block, the cue was reliable and pointed towards the probe (letter) in the probe array on each trial. In the other block, 4 out of 16 trials were congruent, thus making the cue an unreliable indicator of the future location of the probe. Block order was counterbalanced across participants. The test array duration was always 188 ms. The 4 probes appeared in each of the 4 locations to make up the 16 trials in each block.

**Data analysis**

*Performance exclusion criteria*

If participants failed to achieve an above chance accuracy on congruent catch trials, defined by a proportion of correct responses lying outside a 95% binomial confidence interval around *p*=0.25, they were excluded based on task performance.

*Eye data analysis*

Offline, the automated EyeLink procedure was used to identify saccades. Saccades were considered correct if the endpoint was closer to the cued location than to any other stimulus location. Trials were discarded from further analyses if the following criteria were met: no saccade > 1.5 degrees visual angle to the cued location; saccade RT < 80 ms; saccade onset prior to probe array offset; probe array offset > 200 ms before saccade onset.

Saccade kinematics including latency, peak velocity, amplitude, duration, landing site error (distance between saccade endpoint and saccade target center point), and endpoint scatter (the standard deviation of landing site error) were calculated for correct saccades for each group. Kinematics were compared between groups using two-sample t-tests.

*Performance analysis*

The SMART technique first smooths response accuracy over time (with time in this case being the duration between the offset of the probe array and the saccade onset) with a gaussian kernel (standard deviation) of 10 ms, resulting in an average response accuracy per person at each time point. We then performed an arcsine transformation on response accuracy by taking the arcsine of the square root of each value (Snedecor, 1956). Each accuracy value in individual time-courses had a varying amount of data informing the smoothing procedure; therefore, individual response accuracies at each timepoint were associated with a corresponding weight indicating how much data went into the individual accuracy at a given time. At each timepoint, weighted t-tests using the individual accuracies and associated weights were performed to compare responses either between conditions (paired weighted t-tests; van Leeuwen et al., 2019), between groups (two sample weighted t-tests; Shirazi & Huang, 2021), or the differences between conditions between groups (two sample weighted t-tests; Shirazi & Huang, 2021). Significance was determined through permutation testing, with the procedure differing on the basis of the type of test. For the paired t-tests, the condition associated with each response was permuted 10000 times with the smoothing and t-test procedures rerun at each permutation. Across these permutations, null distributions were constructed by summing t-statistics across clusters of adjacent timepoints that passed the significance threshold of p < 0.05. The summed t-statistics of clustered adjacent t-tests passing the significance threshold in the non-permuted dataset that exceeded the 95^th^ percentile of permuted summed t-statistics were considered significant. This approach was used to correct for multiple comparisons and take temporal contingencies into account. A similar approach was used for the group comparisons except the permutation procedure shuffled group membership of trials and the resulting group data were smoothed and submitted to two sample t-tests at each timepoint. The cluster-wise correction procedure remained the same.

**Supplemental Results**

**Eye data**

Trials that did not meet our inclusion criteria were excluded from further analysis. Across all participants there was low rate of excluded trials with a mean rate of 3.64% of trials (SE =0.45%).

Some saccade kinematics (see **Table S1**; latency, landing site error, endpoint scatter, amplitude) did differ between SZ and HC whereas other measures did not (peak velocity, duration).

**Analysis of trials split by long and short probe array duration**

To identify whether the duration of the probe array affected behavior we separated trials based on whether they had long or short probe durations. **Figure S1** shows the main effect of group, the main effect of congruency, and the group differences between congruency conditions for the two probe array durations (118 ms and 188 ms). Group differences collapsed across congruency conditions appear earlier and continue for a longer time in the short probe duration condition (both 195 ms to 140 ms and 138 ms to 77 ms **Figure S1A**) relative to the long probe duration condition (148 ms to 38 ms **Figure S1B**). Trials with a short probe duration show a later congruency effect (beginning 123 ms before saccade onset; **FigureS1C**) relative to the long probe duration trials (beginning 160 ms before saccade onset; **Figure S1D**). On short probe duration trials (**Figure S1E**) a difference between groups in the congruent-incongruent accuracy differences is seen between 172 to 151 ms before saccade onset. This is driven by reduced pre-saccadic attention effects in the SZ group. This difference is not seen in long probe duration trials (**Figure S1F**) where we see no significant differences in the congruent-incongruent accuracy differences between groups.

**Analysis of symptom scores in SZ**

**Figure S2** presents the congruent-incongruent accuracy differences between low and high scorers for each symptom scale, For SANS scores (**Figure S3A**), we observed group differences in the congruent-incongruent accuracy differences driven by larger pre-saccadic effects among participants with symptoms below the median occurring 73 through 58 ms prior to saccade onset. Individual symptoms were not significantly correlated with individual weighted congruent-incongruent accuracy differences averaged over this window (rho=-0.36, p=0.064). For SAPS scores (**Figure S2B**), we observed group differences in congruent-incongruent accuracy differences driven by larger pre-saccadic effects among participants with symptoms above the median occurring 151 through 144 ms prior to saccade onset. Individual symptoms were not significantly correlated with individual weighted differences between accuracy in congruency conditions averaged over this window (rho=0.29, p=0.148).

**Figure S3** presents the results of comparisons of congruency conditions for low and high scorers for each of the symptom scores. To assess where the group differences between congruency conditions arose, we examined congruency condition differences in the above and below median split groups for the SAPS and SANS. The congruency effect in the SANS above median group began later (**Figure S3A**; 115 ms prior to the saccade) than the SANS below median split group (**Figure S3B**; 128 ms prior to the saccade). The congruency effect in the SAPS above median group began earlier (**Figure S3C**; 156 ms prior to the saccade) than the SAPS below median split group (**Figure S3D**; 120 ms prior to the saccade). All congruency effects were significant until the saccade onset.

**Post Test Analysis**

**Figure S4** depicts the results of the post-test. In the reliable cue block we found a non-significant difference in discrimination accuracy t(56) = 1.82, p =.074, such that SZ (M=63.86%, SE = 3.57% ) were marginally less accurate than HC (M=72.84%, SE = 3.39%).

In the unreliable cue block we examined discrimination accuracy using an ANOVA with the factors of cue congruency and group. We found a significant main effect of congruency (F(1,56)=14.32, p<.001), but no significant main effect of group (F(1,56)=1.20, p=.278), or interaction between group and congruency (F(1,56)=0.12, p=.732). The significant main effect of congruency suggests that a congruent cue significantly helped participants identify the probe (M _congruent_ = 66.38%, SE = 5.18%; M _incongruent_ = 52.15%, SE = 3.65%); however, this facilitation appeared to help both groups equally.

Across all congruent trials (in both the reliable and unreliable cue blocks), we examined discrimination accuracy using an ANOVA with the factors of cue reliability and group. We found no significant effects of cue reliability across blocks (F(1,56)=0.33, p=.568), group (F(1,56)=2.31, p=.134), or their interaction (F(1,56)=0.09, p=.763).

**Analysis of CPZ in the SZ group**

**Figure S5** shows the results of a median split analysis for CPZ dosage. The High CPZ group (**Figure S5A**) shows an earlier congruency effect (136 ms prior to the saccade onset) than the Low CPZ group (**Figure S5B**; 82 ms prior to the saccade onset). However, the magnitude of this congruency effect was too small to show a significant difference between median split groups in the difference between congruency conditions (**Figure S5C**). This suggests congruency effects did not differ on the basis of medication dosage.

**Frequency of trials over time**

As a result of the dynamic updating of the probe onset timing per person and over the course of the experiment most trials had a probe array offset within 150 ms preceding saccade onset. The frequency of trials that had probe offsets between 200 to 1 ms before the saccade onset is plotted in **Figure S6**.

**Main Analyses using non-transformed accuracy data**

Analyses of group differences in pre-saccadic attention were conducted on the non-transformed accuracy values and were similar to the results using the arcsine-transformed accuracy values. We found the same effects of group on accuracy when collapsed across congruency conditions (**Figure S7A**): HC were more accurate than SZ in a similar time frame as in the analysis using transformed values. As with the transformed data, HC were more accurate than SZ on congruent trials when the probe array offset was between 200 to 126 ms before saccade onset (**Figure S7B**). Similarly, on incongruent trials (**Figure S7C**), HC were more accurate than SZ between 72 to 144 ms before saccade onset. Collapsed across groups, we found a main effect of congruency condition (**Figure S8A**). While accuracy in the two conditions was initially equivalent, accuracy began to diverge when the probe array offset occurred 154 ms before saccade onset: as the interval between probe array offset and saccade onset decreased, accuracy linearly improved on congruent, but not incongruent trials. This pre-saccadic attention effect was similar between the non-transformed data and the arcsine-transformed data. The accuracy advantage for congruent trials was observed in both groups, but the congruency effect in HC (**Figure S8B**) was present 27 ms earlier than in SZ (**Figure S8C**). This difference was reflected in the analysis comparing congruent-incongruent accuracy differences between the two groups (**Figure S8D**): SZ showed smaller differences between congruency conditions than HC only when the probe offset was 168 to 154 ms before saccade onset.

| Table S1. Saccade Kinematics | | | |  |
| --- | --- | --- | --- | --- |
|  | HC (N = 29) | SZ (N = 29) |  |  |
|  | Mean (SE) | Mean (SE) | Statistic | p-value |
| Latency (ms) | 245 (5) | 273 (8) | t(56)=3.11 | 0.003 |
| Peak velocity (dva/s) | 257 (9) | 241 (11) | t(56)=1.10 | 0.275 |
| Amplitude (dva) | 4.49 (0.10) | 4.15 (0.11) | t(56)=2.29 | 0.026 |
| Duration (ms) | 36 (1) | 38 (1) | t(56)=1.48 | 0.145 |
| Landing site error (dva) | 1.31 (0.06) | 1.52 (0.06) | t(56)=2.51 | 0.015 |
| Endpoint scatter (SD of dva) | 0.715 (0.029) | 0.826 (0.028) | t(56)=2.74 | 0.008 |
| Notes: HC, healthy controls; SZ, individuals with schizophrenia or schizoaffective disorder; dva, degrees visual error; ms, milliseconds; s, seconds; SE, standard error, SD, standard deviation. | | | | |

Figure S1. Short (left) and long (right) probe array duration trials. A) The main effect of group (SZ versus HC) for the short probe array duration when collapsing across congruency conditions. B) The main effect of group (SZ versus HC) for the long probe array duration when collapsing across congruency conditions. C) The main effect of congruency (congruent versus incongruent) conditions for the short probe array duration when collapsing across group. D) The main effect of congruency (congruent versus incongruent) conditions for the long probe array duration when collapsing across group. E) The congruent-incongruent accuracy differences between groups (SZ versus HC) for short probe array duration trials. F) The congruent-incongruent accuracy differences between groups (SZ versus HC) for long probe array duration trials. Smoothed weighted averages with the weighted standard error of the mean are plotted for trials where the probe array offset between 200 to 1 ms before the saccade onset. Across all plots the blue line at the bottom represents clustered significant differences that survived the permutation testing.

Figure S2. Difference between congruency conditions for SZ with above median and below median symptom scores across three symptom scales: A) SANS and B) SAPS. Across all plots the blue line at the bottom of the top row plots represents clusters of significant differences that survived the permutation testing. The error around each line represents the weighted standard error of the mean. In the bottom row the difference in arcsine-transformed weighted accuracy per person is plotted with individual symptom scores.

Figure S3. Congruent versus incongruent trials plotted for the SZ group split based on median symptom scores for SANS and SAPS. A) Congruent versus incongruent trials for SZ with above median SANS scores. B) Congruent versus incongruent trials for SZ with below median SANS scores. C) Congruent versus incongruent trials for SZ with above median SAPS scores. D) Congruent versus incongruent trials for SZ with below median SAPS scores. Smoothed weighted averages with the weighted standard error of the mean are plotted for trials where the probe array offset between 200 to 1 ms before the saccade onset. Across all plots the blue line at the bottom represents clustered significant differences that survived the permutation testing.

Figure S4. Proportion accurate responses for post-test trials in the unreliable and reliable cue blocks where the unreliable block is split into congruent and incongruent trials for HC (on the left) and SZ (on the right).

Figure S5. CPZ equivalent dosage median split in the SZ group. A) Effect of congruency in the above median dosage group. B) Effect of congruency in the below median dosage group. C) Congruent-incongruent accuracy differences between median split dosage groups. Smoothed weighted averages with the weighted standard error of the mean are plotted for trials where the probe array offset between 200 to 1 ms before the saccade onset. Across all plots the blue line at the bottom represents clustered significant differences that survived the permutation testing.

Figure S6. Trial frequency for A) incongruent and B) congruent trials for HC in blue and SZ in red plotted as a function of the probe array offset minus saccade onset.

Figure S7. Group differences in proportion accurate discrimination A) collapsed across congruency conditions, B) in only congruent trials, and C) in incongruent trials. Smoothed weighted averages with the weighted standard error of the mean are plotted for trials where the probe array offset between 200 to 1 ms before the saccade onset. Across all plots the blue line at the bottom represents clustered significant differences that survived the permutation testing.

Figure S8. Congruency differences in arcsine transformed discrimination accuracy A) collapsed across group, B) in only HC participants, and C) in only SZ participants. D) The differences in discrimination accuracy (congruent – incongruent) for the two groups are compared. Smoothed weighted averages with the weighted standard error of the mean are plotted for trials where the probe array offset between 200 to 1 ms before the saccade onset. Across all plots the blue line at the bottom represents clustered significant differences that survived the permutation testing.

**References**

Brainard, D. (1997). The Pyschophysics Toolbox *Spatial Vision*, *10*.

Cornelissen, F. W., Peters, E. M., & Palmer, J. (2002). The Eyelink Toolbox: Eye tracking with MATLAB and the Psychophysics Toolbox. *Behavior Research Methods, Instruments, & Computers*, *34*(4), 613-617. <https://doi.org/10.3758/BF03195489>

Shirazi, S. Y., & Huang, H. J. (2021). Differential theta-band signatures of the anterior cingulate and motor cortices during seated locomotor perturbations. *IEEE Transactions on Neural Systems and Rehabilitation Engineering*, *29*, 468-477.

Snedecor, G. W. (1956). *Statistical methods: applied to experiments in agriculture and biology*. The Iowa state college press.

van Leeuwen, J., Smeets, J. B., & Belopolsky, A. V. (2019). Forget binning and get SMART: Getting more out of the time-course of response data. *Attention, Perception, & Psychophysics*, *81*(8), 2956-2967.
