# Supplementary figures and images for: Pre‐saccadic shifts of attention in individuals diagnosed with schizophrenia
Source: Brain Behav. 2024 Mar 7;14(3):e3466. doi: 10.1002/brb3.3466 (PMC10918725; doi:10.1002/brb3.3466)

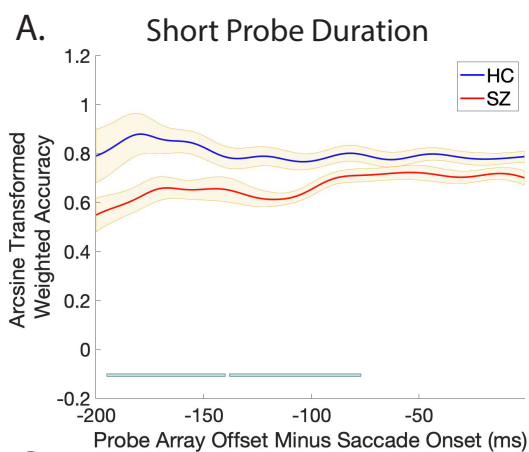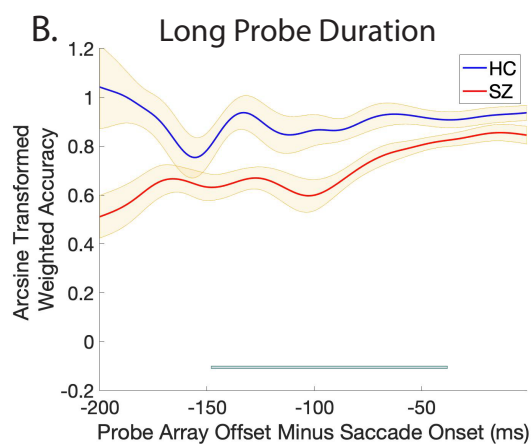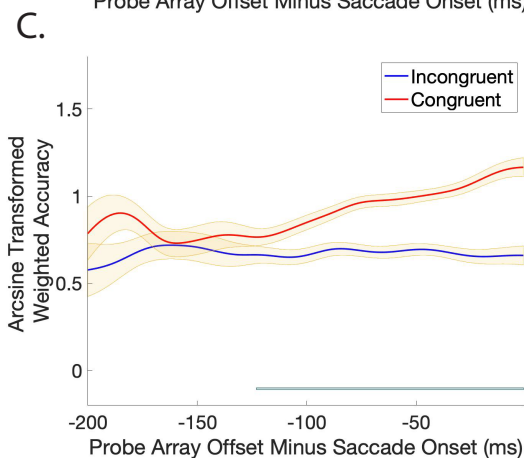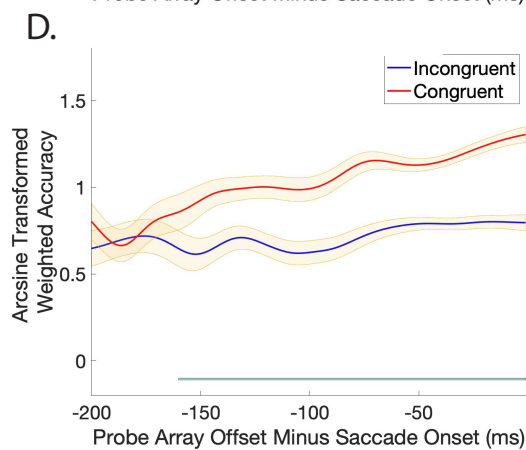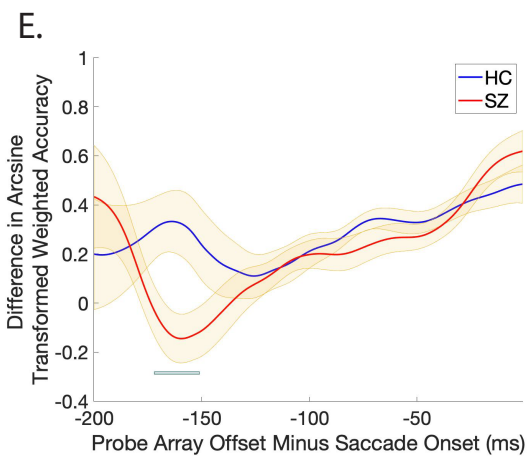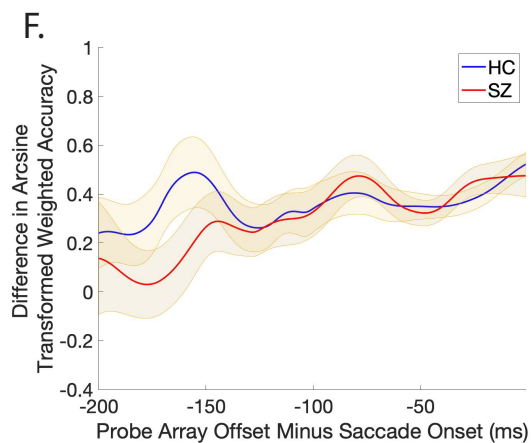

Supplement: Supplementary file 2 — Supporting Information [file BRB3-14-e3466-s005.pdf]

A.

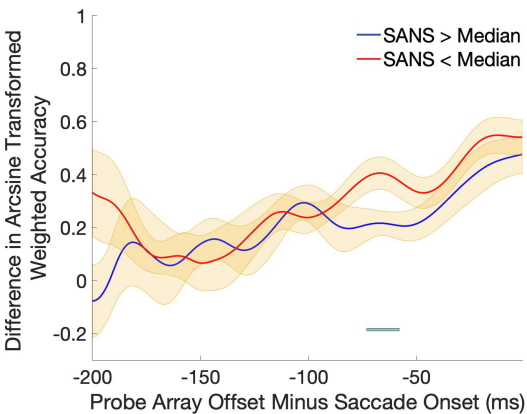

B.

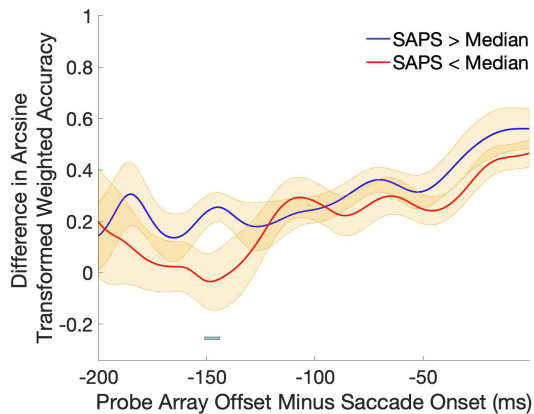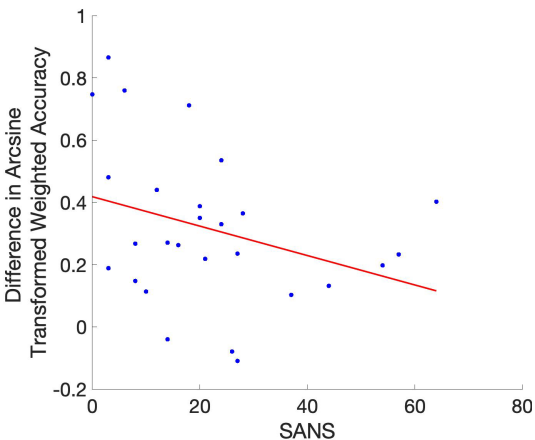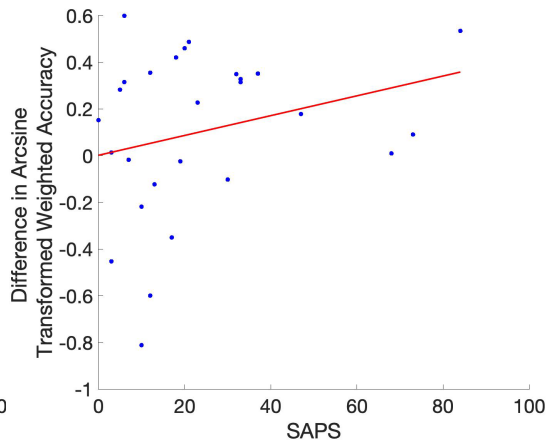

Supplement: Supplementary file 3 — Supporting Information [file BRB3-14-e3466-s006.pdf]

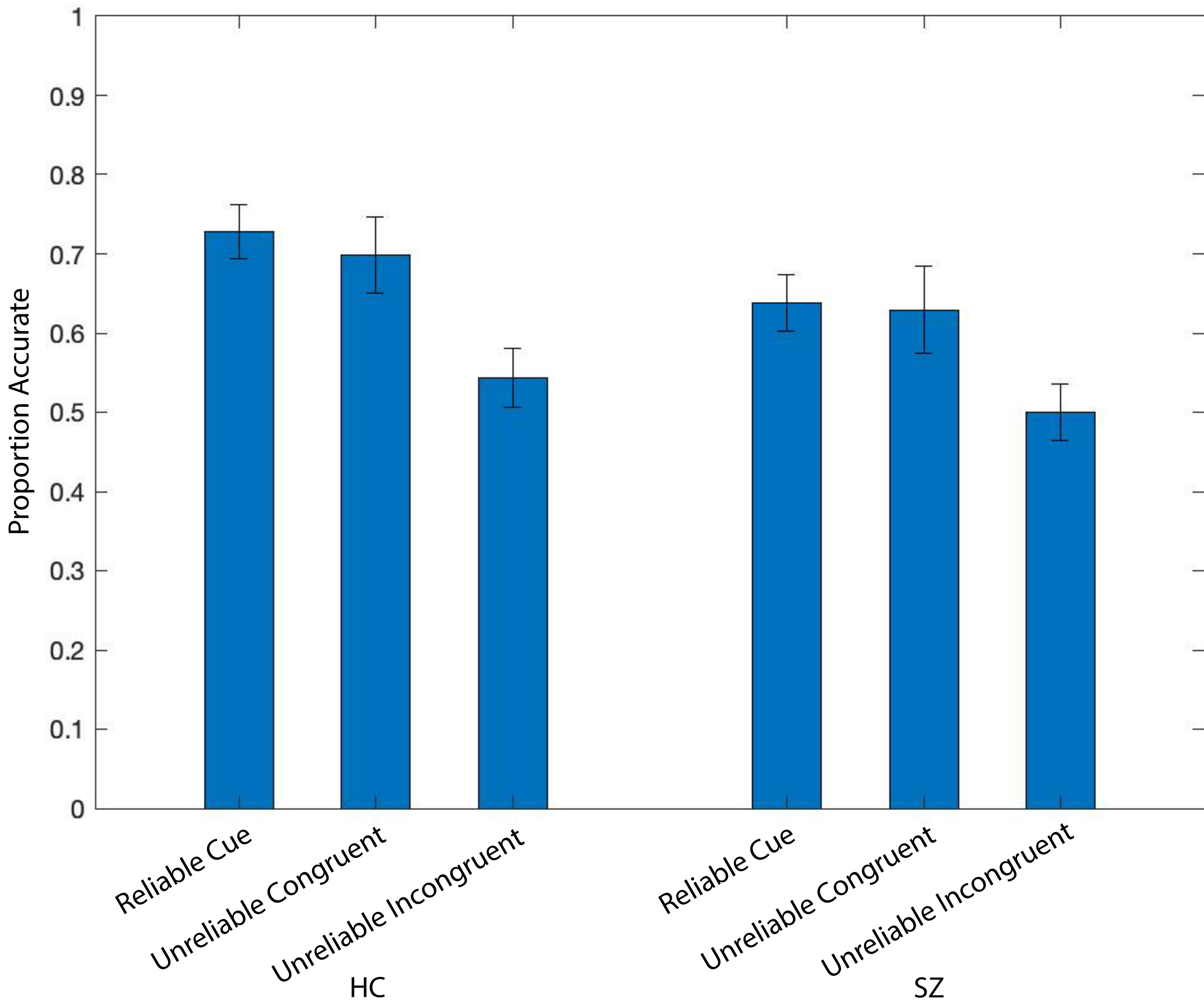

Supplement: Supplementary file 5 — Supporting Information [file BRB3-14-e3466-s003.pdf]

A.

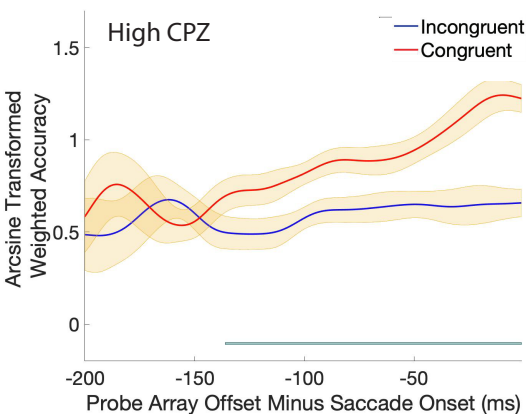

B.

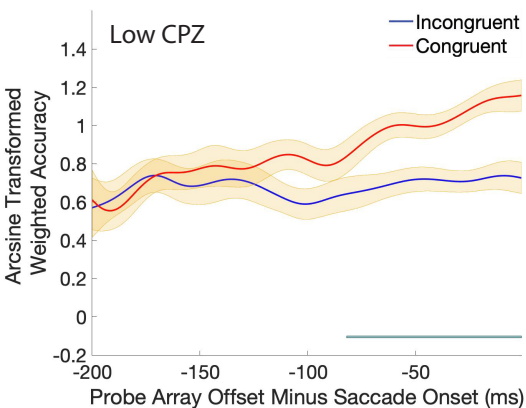

C.

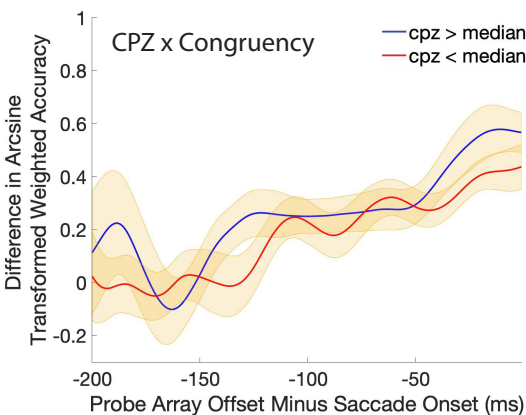

Supplement: Supplementary file 6 — Supporting Information [file BRB3-14-e3466-s007.pdf]

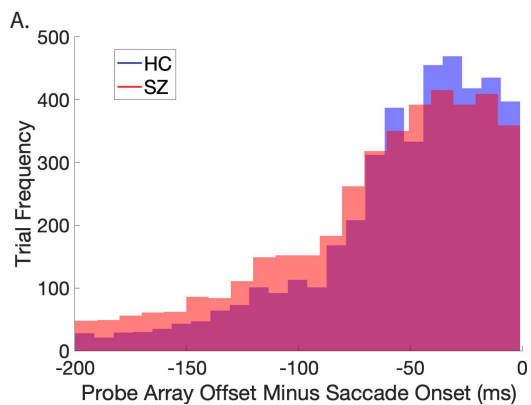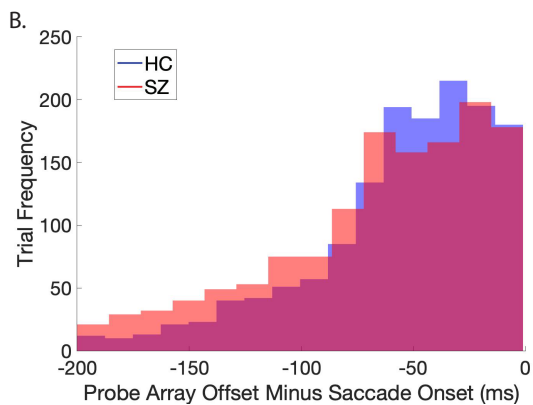

Supplement: Supplementary file 7 — Supporting Information [file BRB3-14-e3466-s004.pdf]

A.

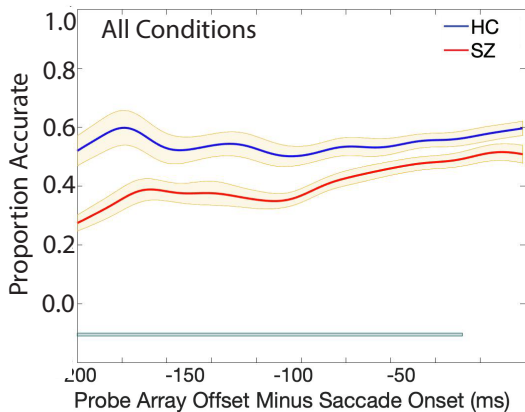

B.

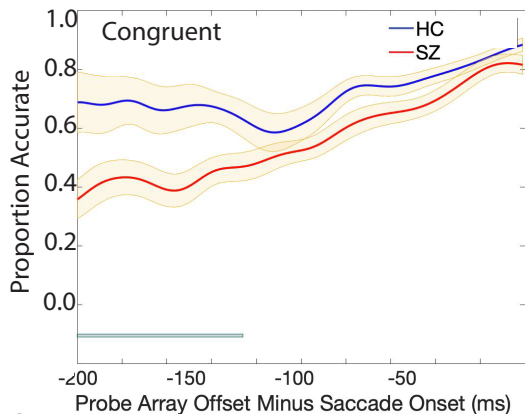

C.

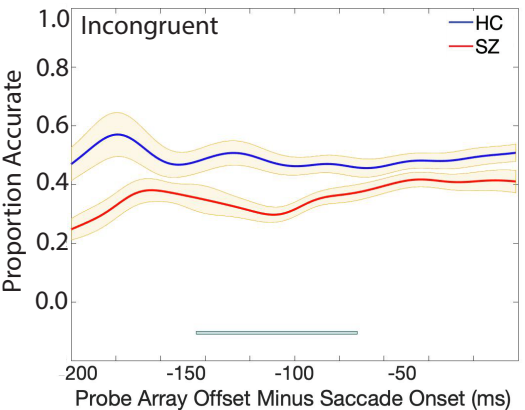

Supplement: Supplementary file 8 — Supporting Information [file BRB3-14-e3466-s008.pdf]

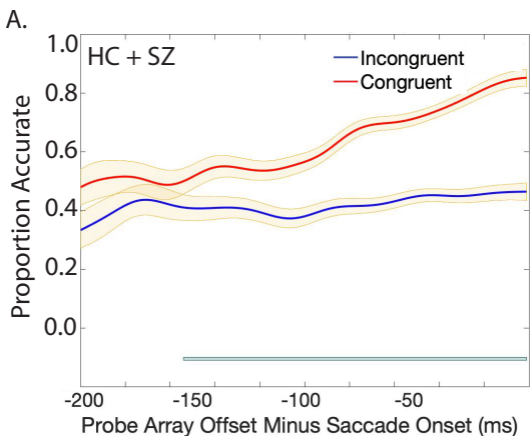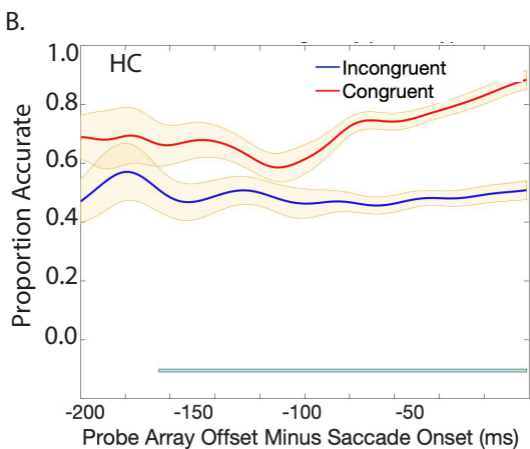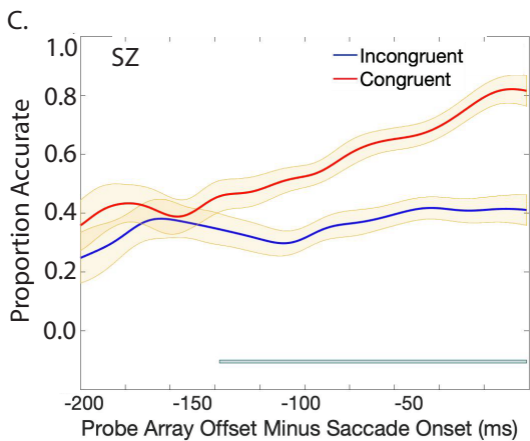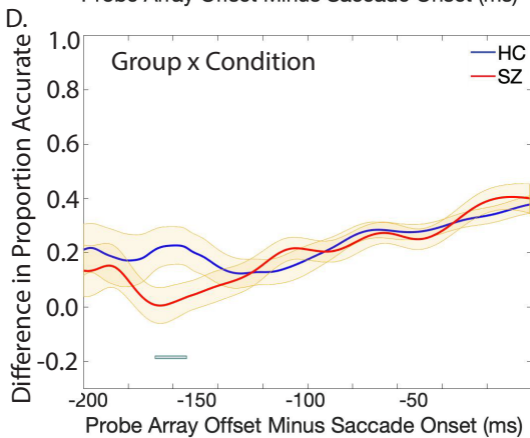

Supplement: Supplementary file 9 — Supporting Information [file BRB3-14-e3466-s009.pdf]
