# Supplementary material for: Pre‐saccadic shifts of attention in individuals diagnosed with schizophrenia
Source: Brain Behav. 2024 Mar 7;14(3):e3466. doi: 10.1002/brb3.3466 (PMC10918725; doi:10.1002/brb3.3466)
Supplement: Supplementary file 4 — Supporting Information [file BRB3-14-e3466-s002.pdf]

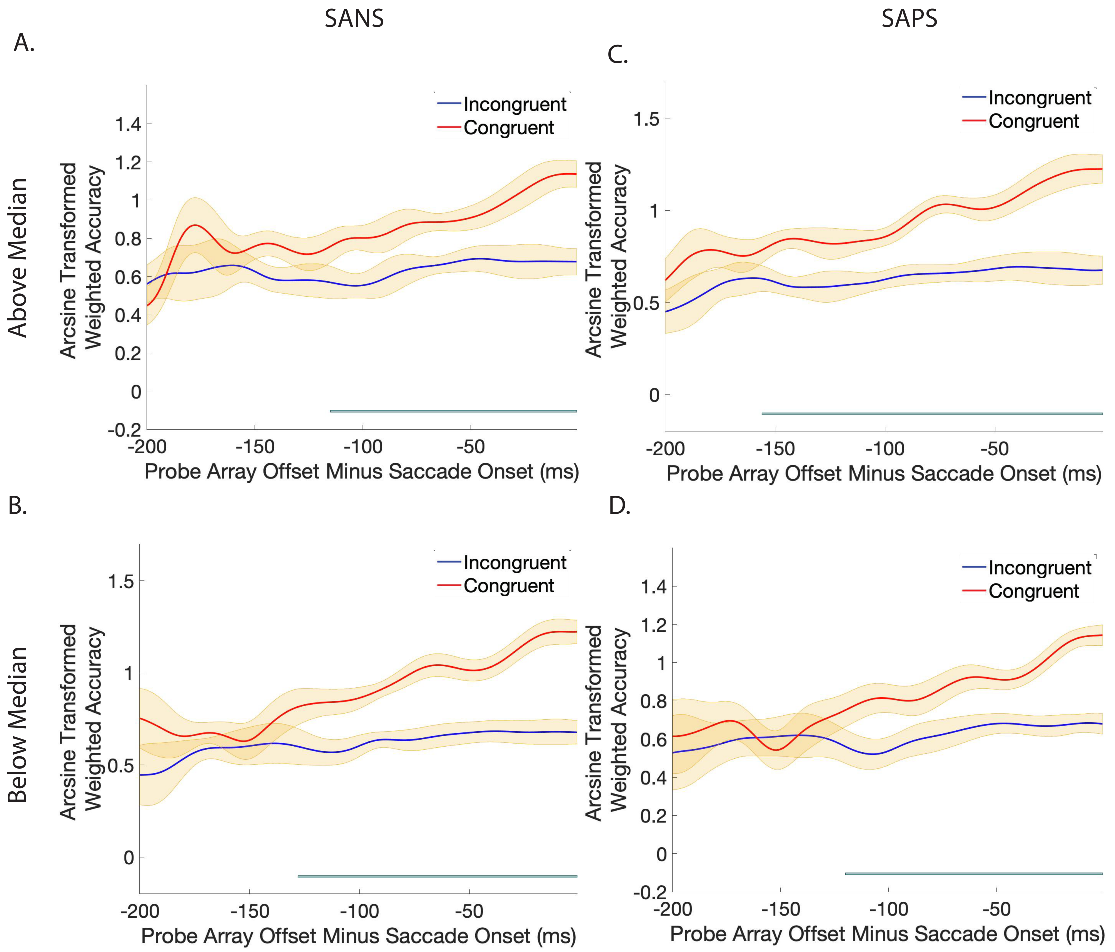
SANS
SAPS
A.
B.
C.
D.
Above Median
Below Median
Arcsine Transformed Weighted Accuracy
Incongruent
Congruent
Probe Array Offset Minus Saccade Onset (ms)
-200
-150
-100
-50
-0.2
0
0.2
0.4
0.6
0.8
1
1.2
1.4
0.5
1.5
